# Turning Symbolic: The Representation of Motion Direction in Working Memory

**DOI:** 10.3389/fpsyg.2016.00165

**Published:** 2016-02-16

**Authors:** Tal Seidel Malkinson, Yoni Pertzov, Ehud Zohary

**Affiliations:** ^1^Department of Neurobiology, Alexander Silberman Institute of Life Sciences, Hebrew University of JerusalemJerusalem, Israel; ^2^Department of Psychology, Hebrew University of JerusalemJerusalem, Israel; ^3^Institut National de la Santé et de la Recherche Médicale U1127, Centre National de la Recherche Scientifique UMR 7225, UMR S 1127, Évaluation Physiologique chez les Sujets Sains et Atteints de Troubles Cognitifs (PICNIC Lab), Institut du Cerveau et de la Moelle Épinière, Sorbonne Universités, Université Pierre et Marie Curie-Paris 06Paris, France; ^4^The Edmond and Lily Safra Center for Brain Sciences, Hebrew University of JerusalemJerusalem, Israel

**Keywords:** visual working memory, motion, representation, working memory

## Abstract

What happens to the representation of a moving stimulus when it is no longer present and its motion direction has to be maintained in working memory (WM)? Is the initial, sensorial representation maintained during the delay period or is there another representation, at a higher level of abstraction? It is also feasible that multiple representations may co-exist in WM, manifesting different facets of sensory and more abstract features. To that end, we investigated the mnemonic representation of motion direction in a series of three psychophysical experiments, using a delayed motion-discrimination task (relative clockwise∖counter-clockwise judgment). First, we show that a change in the dots’ contrast polarity does not hamper performance. Next, we demonstrate that performance is unaffected by relocation of the Test stimulus in either retinotopic or spatiotopic coordinate frames. Finally, we show that an arrow-shaped cue presented during the delay interval between the Sample and Test stimulus, strongly *biases* performance toward the direction of the arrow, although the cue itself is non-informative (it has no predictive value of the correct answer). These results indicate that the representation of motion direction in WM could be independent of the physical features of the stimulus (polarity or position) and has non-sensorial abstract qualities. It is plausible that an abstract mnemonic trace might be activated alongside a more basic, analog representation of the stimulus. We speculate that the specific sensitivity of the mnemonic representation to the arrow-shaped symbol may stem from the long term learned association between direction and the hour in the clock.

## Introduction

Visual working memory (WM) refers to the brief storage of visual information that is used to guide our ongoing actions (Baddeley, 1986; Pasternak and Greenlee, 2005). Clearly, we have an effective representation of the stimulus in WM when it is no longer present. But is that the initial, sensorial (analog?) representation, which persists across the delay period or do we make use of another representation, at a higher level of abstraction?

Some commonalities between visual perception and WM function were demonstrated in several behavioral studies. For example, Kang et al. (2011) have shown that repulsion, the well documented increased perceived *separation* between two simultaneously presented motion directions (Marshak and Sekuler, 1979), is also apparent between a remembered and a just-perceived motion. This seems to hold for oriented Gabors as well (Scocchia et al., 2013). It seems that separate memory storage processes are available for different visual features such as spatial frequency, orientation or speed of motion, mirroring the different processing pathways of these features (Pasternak and Greenlee, 2005).

A joint representation for perception and WM, also implies that the two share a common spatial reference frame, be it in retinotopic coordinates or in another coordinate frame. Accordingly, better discrimination thresholds are reported when remembered and perceived motion directions are in register, either on the retina (Zaksas et al., 2001) or in spatiotopic coordinates, i.e., a reference frame beyond the retinotopic one, be it head-based, body-based or else (Ong et al., 2009).

Traditionally, high level cortical areas were linked to the maintenance of information in WM, amongst which are the prefrontal, temporal and parietal cortices (Larocque et al., 2014; Pratte and Tong, 2014). However, growing evidence from physiological and neuroimaging studies indicates that sensory cortical areas may also be involved in WM and that the same cortical networks participating in visual perception are active during WM (Bisley et al., 2004; Pasternak and Greenlee, 2005; Postle, 2006; Zaksas and Pasternak, 2006; Ester et al., 2009; Silvanto and Cattaneo, 2010; Riggall and Postle, 2012; Emrich et al., 2013; Pratte and Tong, 2014; However, see for example Mendoza-Halliday et al., 2014 for an opposite view). For example, the middle temporal area (MT), one of the key cortical regions in motion processing (Maunsell and Newsome, 1987; Clifford and Ibbotson, 2002), was also implicated in the retention of motion in WM (Pasternak and Zaksas, 2003; Bisley et al., 2004; Pasternak and Greenlee, 2005; Zaksas and Pasternak, 2006; Silvanto and Cattaneo, 2010; Riggall and Postle, 2012; Emrich et al., 2013). Specifically, maintenance of motion information in memory is associated with *transient* activity in MT, which is specific to the remembered stimulus’ features (Bisley et al., 2004; Zaksas and Pasternak, 2006; Riggall and Postle, 2012; Emrich et al., 2013). MT involvement in WM was demonstrated also by Zokaei et al. (2014) who applied Transcranial magnetic stimulation (TMS) to the human analogs regions of MT and the medial superior temporal (MST) (hMT+) during retention of two motion directions while manipulating the task relevance and serial position of the remembered stimuli. They found that only the recall precision of the privileged (i.e., the task-relevant or recent item) motion direction was influenced by TMS over hMT+ (Zokaei et al., 2014). Some studies also show that it is the activity in visual areas that maintains visual information, while the activity in higher brain regions may hold information about other more general aspects related to task demands (Zaksas and Pasternak, 2006; Riggall and Postle, 2012; Emrich et al., 2013; Larocque et al., 2014). For instance, Zaksas and Pasternak (2006) recorded neural responses in monkeys’ prefrontal cortex, and area MT, during a delayed motion discrimination task. They found that neurons in both regions maintained directional responses during the delay period. MT activity during presentation of the Test stimulus typically reflected the *comparison* between it and the remembered sample stimulus. PFC activity reflected more cognitive aspects, such as the task relevance of the motion stimulus, or the forthcoming decision (based on MT cells output). These results suggest that some mnemonic aspects of the motion stimulus are encoded in areas that are active during the initial perceptual stage, but there may well be multiple mnemonic representations of motion direction at various levels of abstraction.

However, the mnemonic representation does not always mimic its perceptual counterpart. Viewpoint-independent mnemonic representation was previously found for biological movement stimuli (Wood, 2010). It was also found that visual WM for sequentially presented patterns was insensitive to spatial displacements, when inter stimulus interval was longer than 100 ms, during fixation (Phillips, 1974) or with an intervening saccade (Irwin, 1991). Correspondingly, the representation in visual WM was shown to be insensitive to spatial properties such as orientation and scale, and therefore may be abstract in nature (Brockmole and Wang, 2003; Brockmole and Irwin, 2005). Further, the divergence between the perceptual and mnemonic representations is also supported by neuroimaging data. For instance, Ester et al. (2009) used fMRI and multivoxel pattern analysis (MVPA) to examine feature-specific activations in early visual regions during memory maintenance. Orientation discrimination based on the multivoxel activation patterns during the delay period (when the stimulus is no longer present) was significantly above chance both in regions of early visual cortex whose receptive fields corresponded to the retinotopic position of the remembered item, and in the *ipsilateral* hemisphere (mapping the opposite visual field). This suggests a spatially global memory representation, that differs from the perceptual retinotopic one (Ester et al., 2009). In a recent fMRI/MVPA study, Pratte and Tong (2014) attempted to reconcile these seemingly controversial results, by testing the hypothesis that early visual areas can maintain information in a spatially specific manner if the task encourages the binding of feature information to a specific location. They found that under such demands, the orientation of the remembered grating was classified more accurately based on activity patterns in the contralateral, than in the ipsilateral hemisphere V1 and V2, suggesting that the spatial specificity of the memory-related activation patterns depends on task demands. Whether this is a general phenomenon, remains an intriguing and open question.

Here we set out to explore the nature of the mnemonic representation of motion direction. In a series of three experiments, we systematically studied factors potentially affecting the retention of motion direction in WM. All experiments incorporated a delayed motion-direction discrimination task in which subjects had to judge whether a perceived moving dot array moved clockwise (CW) or counter-clockwise (CCW) relative to a remembered moving dot array. Specifically, we manipulated contrast polarity (Experiment 1), spatial location specificity (Experiment 2) and introduced pictorial abstract interference during the retention period (Experiment 3) to test if these affect behavioral performance. We find that motion discrimination (when based on comparison with a stored motion signal) is polarity independent, spatially global and prone to pictorial abstract interference, all of which point to the existence of an abstract representation of the motion signal, which characterizes higher-order visual areas. We speculate that this divergence from the initial sensory representation (the two of which might co-exist) may be caused by the particular task demands (CW/CCW judgment) that evoke an abstract representation due to the long-term learned association between direction and the hour in the clock. Thus, these results contribute to the notion of the dependence of memory representation on a specific context.

## Materials and Methods

### Subjects

Sixteen subjects participated in Experiment 1 (mean age 24 ± 4 years, seven males), 13 subjects participated in Experiment 2 (mean age 25.2, five females) and 15 participated in Experiment 3 (mean age 26.5 ± 6, nine males). Nine subjects participated in both Experiments 1 and 3, making a total of 35 subjects. Thirty three of them were naïve and two were experienced subjects. Subjects had normal or corrected visual acuity by self-report. They all gave written informed consent. Experimental procedures were approved by the ethics committee of the Hebrew University of Jerusalem.

### Stimuli and Experimental Settings

Stimuli were videos of moving dots’ arrays, created in MATLAB (MATLAB version 7.7.0.471 Natick, MA, USA: The MathWorks Inc. 2008) and Psychophysics Toolbox (Brainard, 1997). They were shown on a 19-inch CRT monitor (Graphics Series G90fB, View Sonic, Los Angeles, CA, USA, 1024 × 780, 75 Hz) using Experiment Builder software (Experiment Builder, SR Research, Ottawa, ON, Canada). Viewing distance was 64 cm and was enforced using a chin and forehead rest. All experiments were conducted in the dark.

#### Experiment 1

**FIGURE 1:**
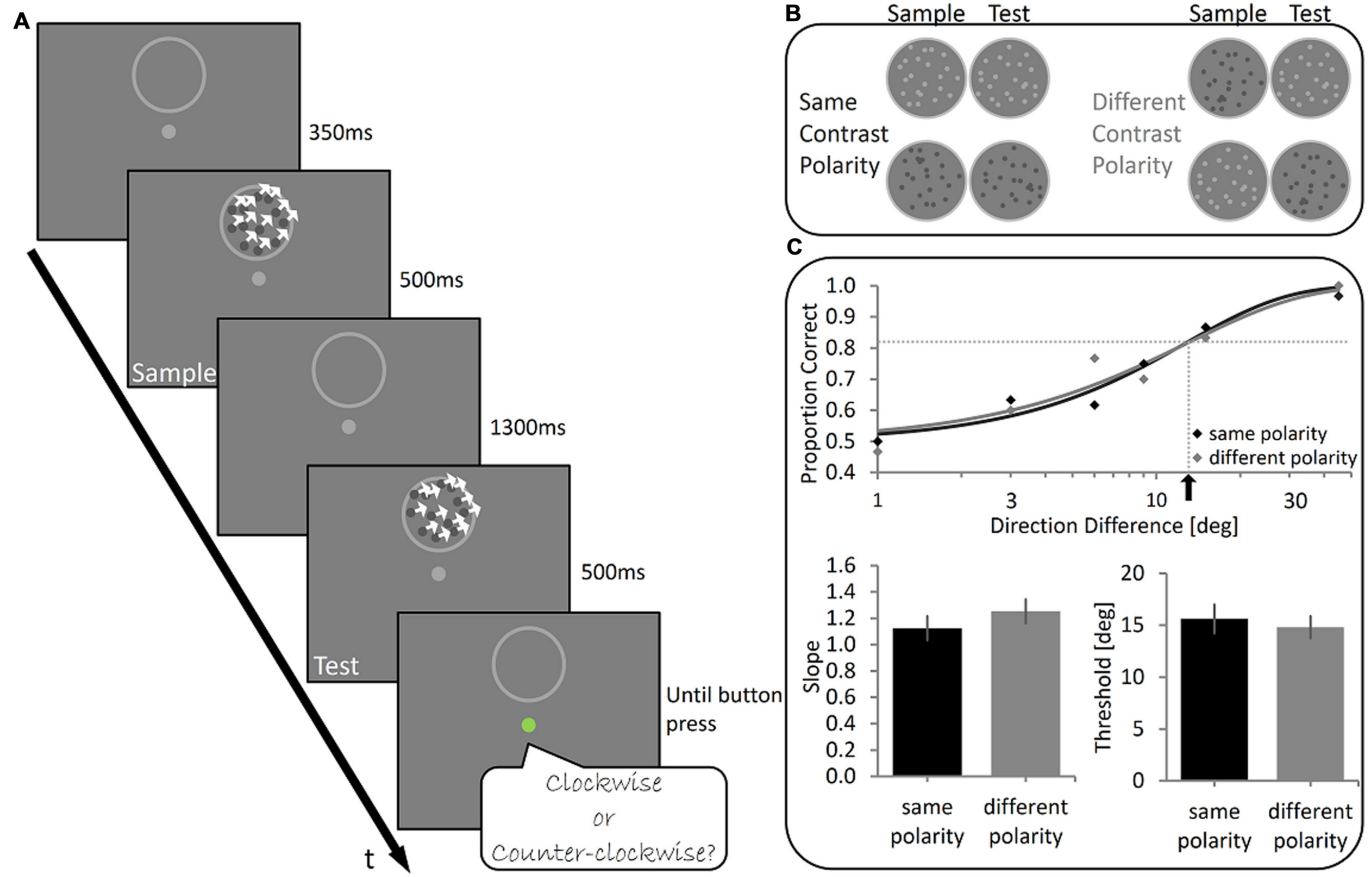
**Experiment 1 – Design and Results (A)**. Illustration of the stimuli used and the trial’s temporal sequence. Two 500 ms-long kinetic random-dot arrays (at 100% coherence) were sequentially presented (Sample and Test stimuli) with an inter-stimulus-interval of 1300 ms between them. Subjects judged whether the direction of motion of the Test stimulus was clockwise (CW) or counter-clockwise (CCW) relative to the direction of motion of the Sample stimulus. **(B)** The experimental conditions. The dots composing the Sample and the Test stimuli had either the same contrast polarity (On Sample-On Test or Off Sample-Off Test) or opposite contrast polarity (On Sample-Off Test or Off Sample-On Test). **(C)** Top panel: Illustrative results of one subject. Data were fitted with a Weibull function and the slope and threshold were calculated (dotted lines and black arrow indicate the threshold). Bottom panel: Group mean results showing no significant difference in the slope (left) and in the threshold (right) between Same Polarity and Different Polarity conditions. Error bars represent SEMs.

Stimuli were arrays of fully coherent moving dots within a circular aperture of 3.5° radius, located 0.5° above fixation (distance from fixation to aperture’s center was 5.3°; **Figure 1A**). The grayscale dots were either light on a darker background or dark on a lighter background (On and Off stimuli respectively), with a michelson contrast of 16.67%. On dots’ luminance was 10.5 cd/m^2^, Off dots’ luminance was 3.4 cd/m^2^ and the background’s luminance was 7.5 cd/m^2^. Dots’ size was 0.8° radius, 100% coherence, dots’ density was 10.2 dot/^∘2^ and dots’ lifetime was uniformly distributed with a 33 ms minimum and a 100 ms maximum.

#### Experiment 2

**FIGURE 2:**
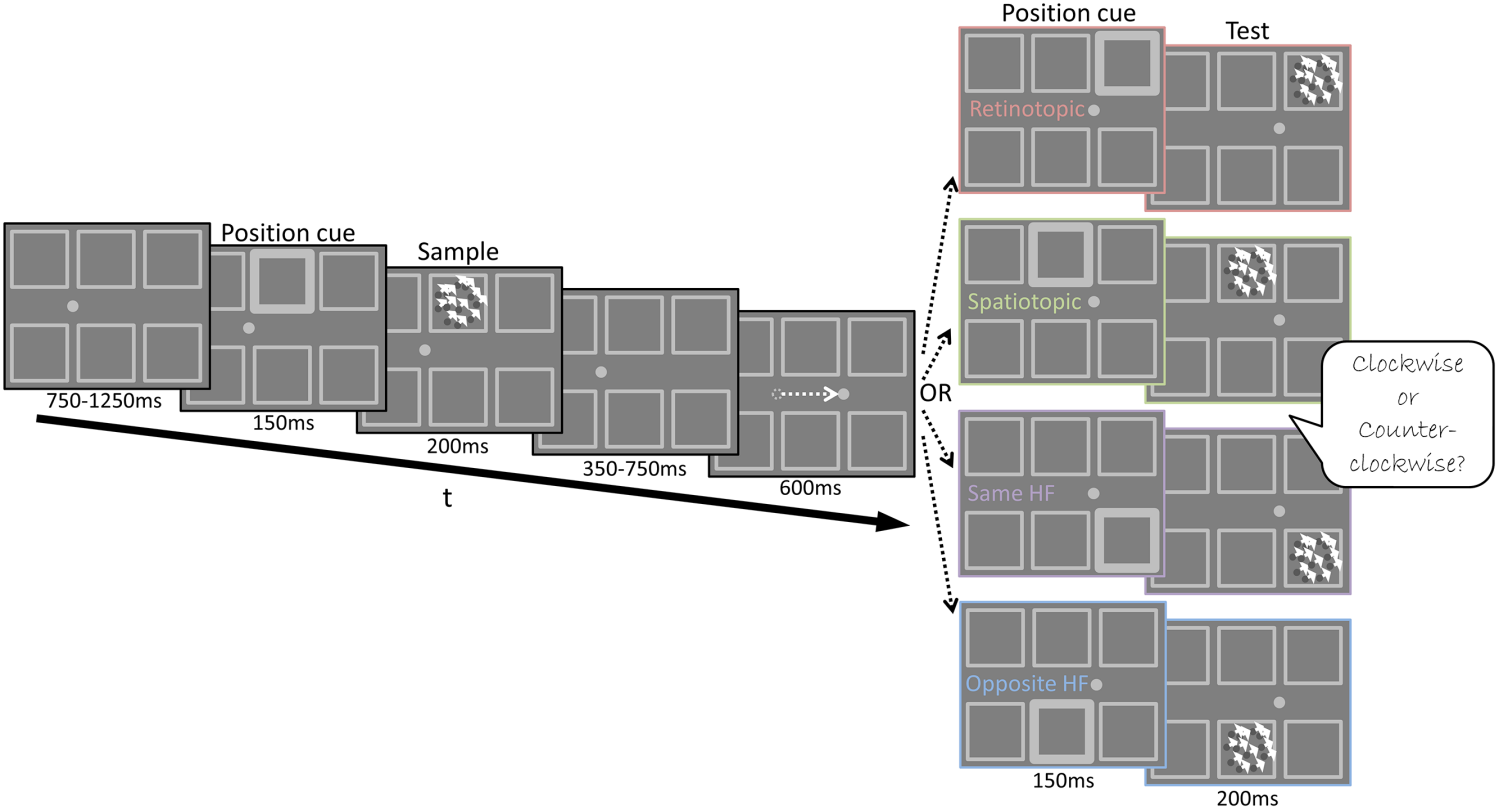
**Experiment 2 Design – Illustration of the stimuli used in Experiment 2 and the trial’s temporal sequence**. A Posner cue (brightened square frame) preceded the presentation of the Sample stimulus. Then the fixation point jumped to its new location, instructing subjects to perform a saccade to its new position. Next, a second Posner cue was shown (600 ms after the new fixation), followed by the Test stimulus. The Test appeared in one of four possible cued locations: same retinotopic location as the Sample but at a different screen location (Retinotopic condition); same screen location as the Sample, yet at a different location on the retina (Spatiotopic condition); different location on the retina and screen but in the same hemifield as the Sample (Same HF condition); different location on the retina and screen in the opposite hemifield to the Sample (Opposite HF condition). Subjects judged whether the direction of motion of the Test stimulus was CW or CCW relative to the direction of motion of the Sample stimulus.

Stimuli were arrays of fully coherent moving dots presented within an array of six 7°-wide rectangular apertures arranged in a 3 × 2 formation, with vertical and horizontal distances of 3.5° between them. Fixation point was presented in two possible locations at a distance of 1.75° from the nearest apertures (fixation to aperture‘s center 5.3°; **Figure 2**). The dots were light on a darker background, with a michelson contrast of 16.67%. All other dot parameters were identical to the ones in the previous experiment.

#### Experiment 3

Light dots on a darker background similar to the Off stimuli from Experiment 1 were used, but with a vacant circular zone (1.2° radius) at the center of the aperture. In the Cue condition, an arrow shaped stimulus was presented (2.4° long, 0.2° wide, luminance and contrast as in Experiment 1) at the aperture’s center. There was no spatial overlap between the moving dots and the arrow stimuli. In the No Cue condition, a circle (1.7° radius, same area, luminance and contrast to the arrow) was shown instead of the arrow stimuli (**Figure 4A**).

### Eye Tracking

A video-based infrared desk-mounted eye tracker (Eye Link1000, SR Research, Ottawa, ON, Canada) with a sampling rate of 1000 Hz was used for recording eye movements. The manufacturer’s software was used for stimuli presentation, calibration, validation, drift correction, and determination of periods of fixation. The eye-position data was used to automatically monitor online performance of the task. Throughout the experiments, deviation from fixation by more than 1.5° to any direction, as well as a failure to execute the required saccade within 600 ms of target presentation in Experiment 2, automatically aborted the trial that was then recycled and returned later on in the block.

### Experimental Design

#### Experiment 1

The temporal sequence was as follows: each trial started with a central fixation point (0.4° diameter; **Figure 1A**). After 350 ms of successful fixation, a 500 ms movie of a fully coherent random dot array (Sample stimulus) appeared. The dots moved translationally in one of 10 different directions (range: 45°–135°; 0° denotes rightward motion) at a 2°/s. Next, the dot array disappeared (leaving only the aperture border visible) and the subject maintained fixation for 1300 ms. Then, a second 500-ms movie of a translationally moving random-dot array (Test stimulus) was presented. The translational direction of the Test stimulus’ motion was CW or CCW relative to the Sample stimulus‘ motion (direction shift range: ±1°–45°; **Figure 1C**). Subjects reported by a mouse press whether the Test stimulus’ direction of motion was CW or CCW, relative to the Sample’s motion direction. There were two experimental conditions: Same Polarity (SP) and Different Polarity (DP). In the SP condition the Sample and the Test stimuli had the same dot-to-background contrast (either On Sample-On Test or Off Sample-Off Test), while in the DP condition the Test stimuli had opposite dot-to-background contrasts (either On Sample-Off Test or Off Sample-On Test). There were 960 trials in total, divided into four sessions, such that each of the six motion direction shifts was repeated 80 times in each condition. The conditions were presented in randomly interleaved mini blocks of 16 trials. To avoid a bias due to end-of-scale conditions (0° and 180°), one hundred catch trials in which the motion direction had a downward component (i.e., between 180° and 360°) were randomly inserted between the experimental trials and were later discarded from the analysis. A drift correction procedure was performed at the beginning of each session and repeated as needed. A failure to maintain fixation during a trial resulted in the recycling of the trial.

##### Training

To acquaint participants with the task, the first session was preceded by a sequence of at least 16 trials, randomly presenting the different experimental conditions, with a constant 45° motion direction shift between Sample and Test stimuli. Training continued until 80% correct responses‘ rate was reached. These trials were excluded from further analysis.

#### Experiment 2

**FIGURE 3:**
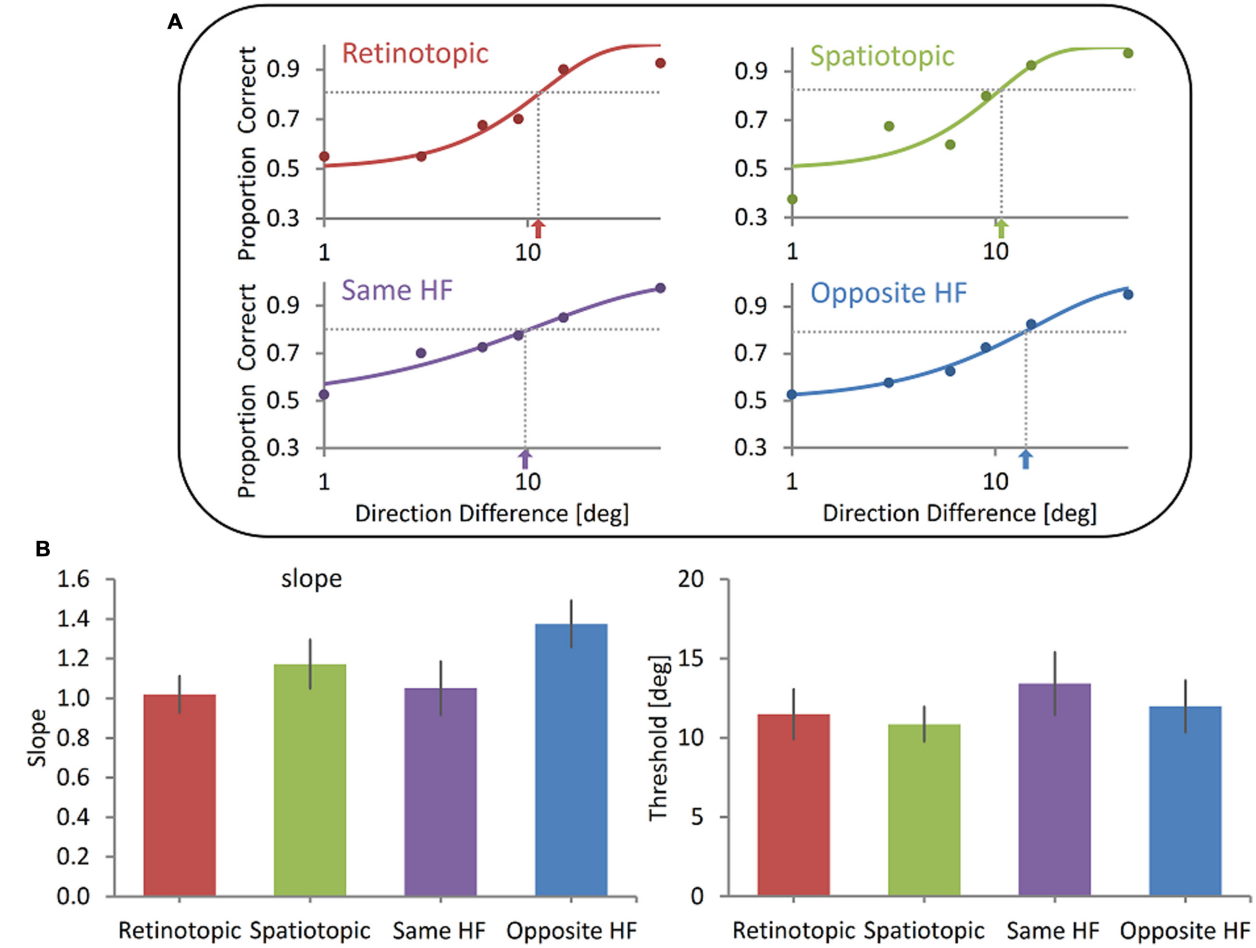
**Spatial invariance of the memory representation of motion direction (A)**. Illustrative results of one subject. Showing no difference between the Retinotopic, Spatiotopic, Same HF and Opposite HF conditions. Data were fitted with a Weibull function and the slope and threshold were calculated (dotted lines and arrows indicate the threshold). **(B)** Group mean results. No significant difference was found between the four conditions either in the slope (left) or in the threshold (right). Error bars represent SEMs.

There were four experimental conditions in Experiment 2: a Retinotopic condition, Spatiotopic condition, Same hemifield, and Opposite hemifield condition (see below). The trials of the four conditions were interleaved randomly and followed the same temporal sequence. First, subjects were presented with a fixation point (0.4° diameter) located 5.3° either to the right or to the left of the screen’s center. After 200 ms of successful fixation, a Posner-like cue (brightened frame) was presented in the middle upper or the middle lower aperture for 150 ms, followed 50 ms later by a 200 ms long video clip of the moving dots array (Sample stimulus), shown in the cued aperture. The dots coherently moved in one of 10 different directions (range: 0°–324°; 0° denotes rightward motion) at a 2°/s. Next, the dot array disappeared (leaving only the apertures borders visible) and the fixation point jumped to its second location, instructing the subjects to perform a saccade (10.6° amplitude) within a 600 ms time window. Then, a second 150 ms long Posner-like cue appeared in one of four possible locations (corresponding to the four experimental conditions, see **Figure 2**): in the same retinotopic location, in the same screen location, in different location within the same hemifield (same HF) or in a different location in the opposite hemifield (opposite HF). Fifty ms later, the Test stimulus appeared in the cued aperture for 200 ms. The direction of the Test stimulus’ translational motion was shifted CW or CCW relative to the Sample stimulus’ motion, by one of six possible direction shifts (±1°–45°; **Figure 3A**). Subjects had to report by a mouse press whether the Test stimulus’ direction of motion was CW or CCW, relative to the Sample’s motion direction. There were 960 trials in total, divided into four sessions, such that each of the six motion direction shifts was repeated 40 times in each condition. A drift correction procedure was performed at the beginning of each session and repeated as needed. A failure to maintain fixation or to correctly perform the saccade within 600 ms resulted in the recycling of the trial.

#### Experiment 3

There were three experimental conditions in Experiment 3: the Oriented Cue condition, the Neutral Cue condition and the No Cue condition.

##### Oriented Cue condition

**FIGURE 4:**
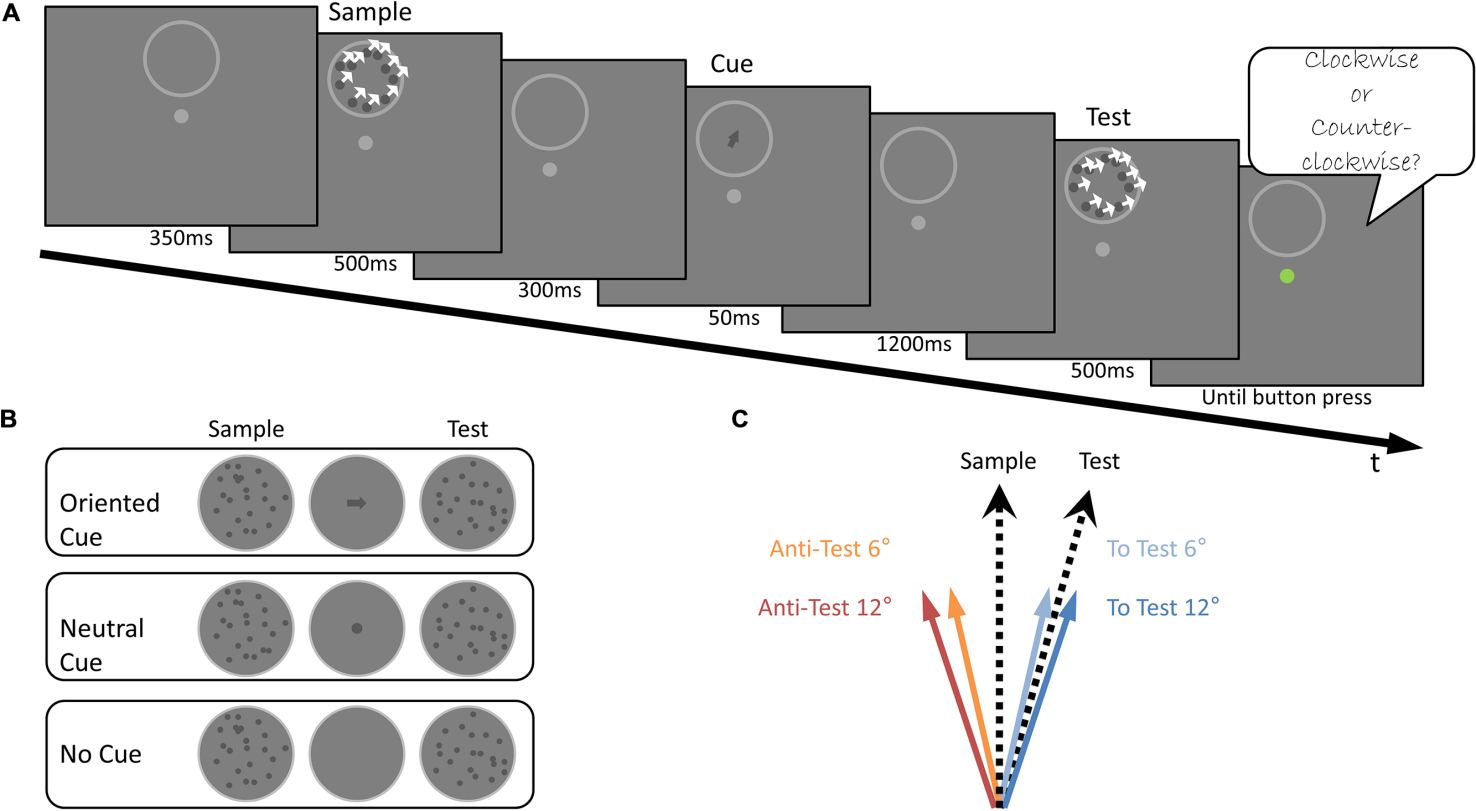
**Experiment 3 Design (A)**. Illustration of the stimuli used and the trial’s temporal sequence. The Sample stimulus was presented, followed by the presentation of a cue for 50 ms, after which the Test stimulus was shown. Subjects were asked to judge whether the direction of motion of the Test stimulus was CW or CCW relative to the direction of motion of the Sample stimulus, ignoring the cue. **(B)** An illustration of the cues used in the three experimental conditions: Oriented Cue, Neutral Cue and No Cue conditions. **(C)** The four arrow orientations used in the Oriented Cue condition. These were shifted from the Sample motion direction, either toward the Test stimulus: To Test 6° (light blue) and To Test 12° (blue), or away from it: Anti-Test 12° (red), Anti-Test 6° (orange).

The temporal sequence was as in Experiment 1 with the following differences: The Sample stimulus’ direction of motion was randomly chosen from 20 possible directions (16°–168°, in steps of 8°). A vacant circular zone without dots remained at the center of the aperture. Two hundred ms after the offset of the Sample stimulus, an arrow was presented for 50 ms, at the center of the aperture (such that there was no spatial and temporal overlap between the Sample and the arrow). The arrow was oriented to one of four possible orientations (**Figure 4C**): shifted by 12° relative to the Sample stimulus’ direction away from the upcoming Test stimulus‘ direction (12° Anti-Test); shifted by 6° relative to the Sample stimulus’ direction away from the upcoming Test stimulus‘ direction (6° Anti-Test); shifted by 6° relative to the Sample stimulus’ direction toward the upcoming Test stimulus‘ direction (6° To Test); and shifted by 12° relative to the Sample stimulus’ direction toward the upcoming Test stimulus’ direction (12° To Test). Next, the arrow disappeared and the subject maintained fixation for 1100 ms. Then, the Test stimulus appeared for 500 ms, its translational direction of motion was always ±9° relative to the Sample stimulus. Subjects had to report by a mouse press whether the Test stimulus’ direction of motion was CW or CCW, relative to the Sample’s motion direction and were instructed to ignore the cue. There were 240 trials in total, divided into 2 sessions. Trials of the four possible arrow orientations were randomly presented. To prevent usage of the arrow as a stimulus replacing the Sample stimulus, half of the trials were of the 12° To Test orientation (since the test was always shifted 9° relative to the Sample, an answer based on the arrow in this case would always yield a wrong answer). The rest of the trials were equally divided between the other three arrow orientations. Sixteen catch trials in which the motion direction was between 180° and 360° were randomly presented between the experimental trials and were later discarded from analysis. A drift correction procedure was performed at the beginning of each session and repeated as needed. A failure to maintain fixation resulted in the recycling of the trial.

##### Neutral Cue condition

Procedure was identical to the Oriented Cue condition, except for the cue being a circle, instead of an oriented arrow (with equivalent contrast and surface). There were 80 trials in total.

##### No Cue condition

Procedure was identical to the Oriented Cue condition except that there was no cue in the 1300 ms delay period between Sample and Test stimuli presentation. There were 80 trials in total.

##### Training

Subjects, who had not participated in Experiment 1, went through a training procedure, similar to that preceding Experiment 1, with no cues. The results were discarded.

### Data Analysis

All data were analyzed using SPSS for Windows (Rel. 16.0.1. 2007, SPSS Inc., Chicago, IL, USA) and Microsoft Excel (2013).

#### Experiments 1 and 2

In order to simplify the comparison of our results with previous studies (e.g., Ong et al., 2009), data were fitted with a Weibull function (frequency of *correct* responses for each motion direction shift; **Figures 1C** and **3A**) using the following formula:

$$f\left(x\right)=1-0.5\,e^{-{\left(\frac{x}{\alpha }\right)}^{\beta }}\,\;\;\;\;\;\;.$$

The goodness of fit of the data to the assigned curve was determined by calculating the model’s explained variance (*R*^2^):

$$R^{2}=1-\frac{u\,n\,\exp l\,a\,i\,n\,e\,d\,\;\;\mathrm{var}\,i\,a\,n\,c\,e}{t\,o\,t\,a\,l\,\;\;\mathrm{var}\,i\,a\,n\,c\,e}=1-\frac{s\,u\,m\,\;\;o\,f\,\;\;s\,q\,u\,a\,r\,e\,s_{d\,a\,t\,a\,\;\;t\,o\,\;\;f\,i\,t}}{s\,u\,m\,\;\;o\,f\,\;\;s\,q\,u\,a\,r\,e\,s_{d\,a\,t\,a\,\;\;t\,o\,\;\;a\,v\,e\,r\,a\,g\,e}}$$

We analyzed data from subjects whose performance was well matched by the Weibull function in every experimental condition (*n* = 15 in Experiment 1; *n* = 10 in Experiment 2, *R*^2^ > 0.8; explaining more than 80% of the variance). Three subjects were excluded *post hoc* from Experiment 2 because their data failed to reach the demanded goodness of fit.

Performance threshold was the estimated α parameter of the Weibull function (the direction shift corresponding to performance of 82%), while the slope was the β parameter.

#### Experiment 3

After verification of the data’s Gaussian distribution and equal variance (Shapiro–Wilk Tests: To Test 12°: *W* = 0.94, *p* = 0.42; To Test 6°: *W* = 0.97, *p* = 0.85; Anti-Test 6°: *W* = 0.90, *p* = 0.09; Anti-Test 12°: *W* = 0.96, *p* = 0.66; Neutral Cue: *W* = 0.97, *p* = 0.92; No Cue: *W* = 0.95, *p* = 0.56; Mauchly’s test of sphericity: Mauchly’s *W*_(14)_ = 0.186, *p* = 0.126), individual Sensitivity Indices (d′) and criterion indices (λ_center_) were calculated for the No Cue condition, the Neutral Cue condition and for the four arrow orientations in the Oriented Cue condition.

$$d^{\prime }=z_{\left(F\,a\,l\,s\,e\,\;\;A\,l\,a\,r\,m\,s\right)}-z_{\left(H\,i\,t\,s\right)}$$

$$\lambda_{c\,e\,n\,t\,e\,r}=-0.5\times \left[z_{\left(F\,a\,l\,s\,e\,\;\;A\,l\,a\,r\,m\,s\right)}+z_{\left(H\,i\,t\,s\right)}\right]$$

Individual Δ d′ scores were calculated by subtracting the participant’s d′ in the Neutral Cue condition from his d′ scores in each of the four arrow orientations.

#### Bayes Factors Analysis

Bayes Factors were computed using the JASP software (Love et al., 2015).

A JZS Bayes Factor repeated measures ANOVA with default prior scales was used in Experiments 2 and 3. Additionally, in Experiments 1 and 3 Bayesian paired sample *t*-tests were performed using a Cauchy prior width of 1.3. Cauchy prior widths of 0.707 and 1.3 were used in Experiment 2 to test for differences in the threshold and slope, respectively. A Gaussian quadrature integration routine was used in both cases.

## Results

### Experiment 1

Subjects performed a delayed motion discrimination task, judging if a random-dot array (Test stimulus) moved CW or CCW relative to a previously presented random dot array (Sample stimulus; **Figure 1A**). The random dots were either all lighter or darker than the background (On and Off conditions, respectively). In the SP condition, the brightness polarity of the Sample and Test stimuli was the same (either On Sample-On Test or Off Sample-Off Test). In the DP condition Sample and Test stimuli had opposite polarity (either On Sample-Off Test or Off Sample-On Test, see **Figure 1B**). A difference in performance between these two conditions would suggest that the direction of motion is encoded and compared in a manner in which brightness polarity is still relevant, typical of low-level visual processing stages in which motion is encoded separately along the On and Off pathways (Clifford and Ibbotson, 2002). However, motion discrimination was virtually the same in both conditions. Illustrative psychometric functions of one participant for both conditions are shown in **Figure 1C**. Across subjects, the mean discrimination threshold was 15.7° (*SD* = 5.7) in the SP condition and 14.8° (*SD* = 4.4°) in the DP condition. Discrimination thresholds in the two conditions did not differ significantly (Wilcoxon Signed-ranks test, *Z* = -1.19, *p* = 0.23). Wilcoxon Signed-ranks test also showed there were no significant differences between the slope in the SP condition (*Mean* = 1.12, *SD* = 0.38) and that in the DP condition (*Mean* = 1.25, *SD* = 0.38), *Z* = 1.02, *p* = 0.31. Thus, we did not reject the null hypothesis that the delayed judgment of motion direction is unhampered by brightness polarity, which selectively activates two separate populations of neuronal pathway in the initial analysis stages of the visual signal (i.e., On or Off pathway).

To further validate these results we performed a Bayesian paired sample *t*-test testing threshold and slope measurements in the SP and DP conditions. For the threshold the test revealed a Bayesian Factor of 0.31 (error% = 1.19e-4), supporting moderately the null hypothesis over H1, i.e., that there is no effect of polarity on delayed direction discrimination threshold. Similar results were found for the slope, with a Bayesian Factor of 0.30 (error% = 1.190e-4), supporting moderately the null hypothesis over H1, i.e., that there is no effect of polarity on the sensitivity to direction in WM.

Thus, performance does not seem to be determined by constraints set by low-level visual processing, but rather more likely to be based on computation in regions in which information from the separate On and Off pathways has been merged.

We continued exploring the memory representation of motion direction and tested its spatial characteristics. Specifically, in Experiment 2 we asked whether the trace is spatially specific, and if so, is it represented in retinotopic or in spatiotopic coordinates.

### Experiment 2

Subjects performed a delayed motion discrimination task, judging if the Test stimulus moved CW or CCW relative to the Sample stimulus (**Figure 2**) in four different conditions. Using an intervening saccade between Sample and Test presentation, we tested performance when the Sample and Test were presented in the same retinotopic location but in different locations on the screen (Retinotopic condition); when the Sample and Test were presented in the same position on the screen but in different locations on the retina (Spatiotopic condition); when the Sample and Test were presented in different locations but in the same hemifield (Same HF condition); when Sample and Test were presented in different locations in opposite hemifields (Opposite HF condition). We reasoned that if the mnemonic trace is spatially specific in a particular coordinate frame, performance will be better when the Sample and Test are presented in matching locations at that coordinate system. As the retinotopic receptive field size progressively increases at successively higher levels in the processing hierarchy (Smith et al., 2001), a retinotopic advantage may indicate that this task is based on the output of a cortical region with receptive fields smaller than 3.5° at 5°eccentricity (the separation between adjacent apertures in our experimental display).

However, we could not reject the null hypothesis that there is no spatial specificity, neither retinotopic nor spatiotopic. Illustrative psychometric functions of one participant for all conditions are shown in **Figure 3A**. Across subjects, the mean discrimination threshold was 11.5° (*SD* = 4.9) in the Retinotopic condition, 10.59° (*SD* = 3.5) in the Spatiotopic condition, 13.4° (*SD* = 6.2) in the Same HF condition and 12.0° (*SD* = 5.2) in the Opposite HF condition (**Figure 3B**). Discrimination thresholds in the four conditions did not differ significantly (Friedman Test, χ^2^_(3,_ *_N_*_=__10)_ = 2.8, *p* = 0.43). Friedman Test also showed there were no significant differences between the slopes in all four conditions (Retinotopic slope *Mean* = 1.02, *SD* = 0.29; Spatiotopic slope *Mean* = 1.17, *SD* = 0.39; Same HF slope *Mean* = 1.05, *SD* = 0.42; Opposite HM slope *Mean* = 1.38, *SD* = 0.37; **Figure 3B**), χ^2^_(3,_ *_N_*_=__10)_ = 3.2, *p* = 0.36. These data suggests that the delayed judgment of motion direction is insensitive to changes in the stimulus’ retinotopic or spatiotopic location. Moreover, direction discrimination does not seem to be affected even when the Sample and Test stimuli are shown in opposite hemifields.

To further validate these results we conducted Bayesian factor analysis. Based on a study by Ong et al. (2009), that found an advantage for direction discrimination when stimuli were displayed on the same spatiotopic location compared to the retinotopic location, we decided to test the hypothesis that the discrimination sensitivity in our experiment followed the same pattern. Thus: if threshold of the psychometric function when stimuli share their Spatiotopic location is lower than when the stimuli share the same Retinotopic threshold. Due to lack of prior knowledge (the slope was not tested in Ong et al., 2009) we did not assume any direction of effect and tested the hypothesis that the Spatiotopic slope differs from the Retinotopic slope. Bayesian paired sample *t*-tests testing threshold and slope in the Retinotopic and Spatiotopic conditions were done with a default Cauchy prior widths of 0.707 for the threshold test and 1.3 for the slope test (because of the lack of prior information on effect size for the latter). For the threshold the test revealed a Bayesian Factor of 0.18 (error % = 4.18e-5), supporting moderately the null hypothesis over H1, i.e., that there is no difference between Retinotopic and Spatiotopic conditions on delayed direction discrimination threshold. Weaker evidence was found when testing the slope, with a Bayesian Factor of 0.44 (error % = 1.85e-6), providing anecdotal support for the null hypothesis over H1.

In addition, a JZS Bayes Factor repeated measures ANOVA testing the threshold scores of the four possible locations (Retinotopic, Spatiotopic, Same HF, Opposite HF) revealed that the null model was preferred to the main effect of location by a Bayes Factor of 0.74 (error % = 0.40). The data provide marginal evidence for the hypothesis that the location of the Sample and Test stimuli does not affect the delayed direction discrimination threshold. For the Slope scores not enough evidence was found to support the null model or the main effect model (Bayes Factor = 1.03, error% = 0.44).

Thus, performance is not limited by spatial constraints that characterize early visual processing (in which neurons have rather small receptive fields). The results suggest that performance is based on computation in regions in which information from big portions of the visual field has been combined to yield a representation that is spatially invariant.

Until now, we have shown that he delayed motion comparison task does not seem to be sensitive to low-level changes in the visual stimulus (e.g., brightness polarity, retinotopic location). These result suggests that judgment might be based on an abstract representation of motion direction. Our third experiment was designed to test this issue directly by presenting a symbolic stimulus (e.g., an arrow that was oriented in various directions) between the sample and test stimuli. The rationale was that if the sample direction of motion is encoded in an abstract fashion, an oriented arrow (which is often used to symbolize motion direction), is likely to affect the represented motion in a predictive fashion.

### Experiment 3

Subjects performed a delayed motion discrimination task, judging if the Test stimulus moved CW or CCW relative to the Sample stimulus (**Figure 4A**) in three possible configurations (**Figure 4B**). In the Oriented Cue condition an arrow-shaped cue, (in one of four possible orientations relative to the Sample stimulus) was briefly presented between the Sample and Test stimuli (**Figure 4C**). We hypothesized that if the memory trace of the Sample’s motion direction is represented in a symbolic form, the motion direction memory trace is likely to be biased toward the arrow’s orientation. Specifically, this arrow-based bias would increase or decrease the motion direction difference between the Sample trace and the upcoming Test stimulus direction (according to the orientation of the arrow with respect to the Test stimulus). Consequently, it would improve or hamper task performance, respectively.

In order to rule out effects resulting from merely having an attention-capturing intervening stimulus between Sample and Test stimuli, we also included a Neutral Cue condition, in which a non-oriented stimulus (e.g., a circle) was briefly presented at the same location as the oriented cue, (i.e., at the center of the dots’ aperture, without spatial overlap with them) between the Sample and Test presentation. Finally, a No Cue condition, in which only the fixation point and aperture border were present between the Sample and Test stimuli, was used to assess baseline performance.

#### Oriented Cue Condition

**FIGURE 5:**
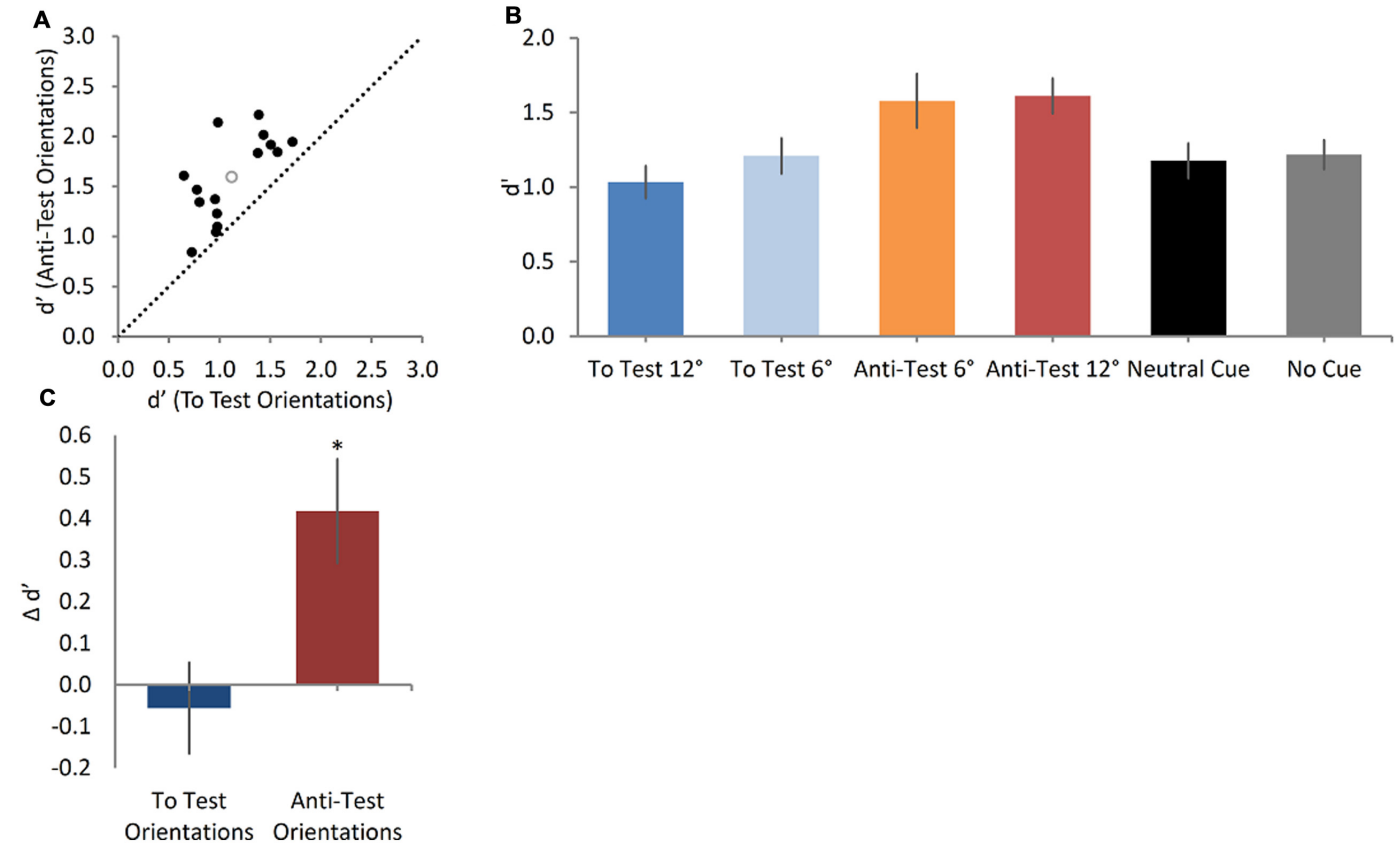
**Cue orientation modulates the sensitivity to motion direction in WM (A)**. A scatter plot showing sensitivity indices (d′) in the Anti-Test orientations and in the To Test orientations (black full circles represent individual data; empty gray circle represents the group mean sensitivity index). The sensitivity to motion direction in the Anti-Test orientations is significantly greater than that in the To Test orientations (Wilcoxon signed-ranks test, *p* = 0.001). **(B)** Mean sensitivity index results for the four types of arrows in the Oriented Cue condition, the Neutral Cue condition and the No Cue condition. Error bars represent SEMs. The orientation of the arrow cue had a significant effect (Friedman test, *p*< 0.001) on the sensitivity to motion direction, in a significant ascending trend (Page’s L test, *p* = 0.039). **(C)** The difference in sensitivity between the Oriented Cue condition and the Neutral Cue condition (serving as a baseline), assessed separately for the Anti-Test and the To-Test orientations. Only the sensitivity in the Anti-Test orientations differed significantly from that in the Neutral Cue condition (One sample *t*-test, *p* = 0.002).

Our results indicate that the symbolic cue had influenced motion judgment in a predictable manner. To test if the Sample’s motion memory trace was attracted to the arrow direction, we analyzed the sensitivity difference (measured in d′) between the Anti-Test-oriented and the To-Test oriented arrows. A Wilcoxon Signed-ranks test indicated that subjects were significantly more sensitive when the arrow was in the Anti-Test-orientations (*Mean* = 1.59) than in the To-Test orientations (*Mean* = 1.12), *Z* = 3.41, *p* = 0.001, *r* = 0.88; (**Figure 5A**). These results confirmed that task performance was better when the arrow was oriented away from the Test stimulus, thus increasing the Sample-Test direction difference, than when it was oriented toward the Test’s direction, thus decreasing that difference. Hence, these data may support the idea that the direction of the Sample is transformed in WM to a higher level abstract representation, which can be modulated by a symbolic cue such as an arrow. This result was confirmed using a Bayesian paired sample *t*-test indicating an extreme support for the hypothesis that subjects were more sensitive when the arrow was in the Anti-Test orientations than in the To-Test orientations (Bayes factor = 1113, error % = ∼1.910e-9).

To further examine the cue’s gradual influence on the discrimination sensitivity we looked separately at the four arrow orientations in the main Oriented Cue condition, and found that the discrimination of motion direction significantly differed between the four orientations (Friedman Test, χ^2^_(3,_ *_N_*_=__15)_ = 18.0, *p* < 0.001; See **Table 1** for Sensitivity index Means and SD, **Figure 5B**). The lowest discrimination performance occurred when the arrow cue was shifted away from the Sample toward the Test direction (To Test 12°), while the best motion sensitivity was when the arrow was pointed in the other direction (Anti-Test 12°), away from the Test. This is further strengthened by a significant ascending trend found in Page’s L test [*L* = 398, χ^2^(1) = 4.2, *p* = 0.039], showing that as the arrow orientation shifted away from the Test, the sensitivity increased. This suggests that the remembered Sample motion direction is biased toward the arrow direction (i.e., causing an “attraction” effect). Bayes Factor repeated measures ANOVA testing the d’ scores of the four possible cues revealed that the model containing the cuing main effect was preferred to the null model by a Bayes Factor of 20.78 (error % = 0.44), providing strong evidence for the hypothesis that the cue orientation affected the sensitivity to motion direction in WM.

**Table 1:** Mean d′ and decision criteria in the four cue orientations.

Cue orientation	d′ Mean (*SD*)	Criterion mean (*SD*)
Anti-Test 12°	1.61 (0.46)	0.04 (0.29)
Anti-Test 6°	1.58 (0.70)	-0.07 (0.39)
To Test 12°	1.21 (0.46)	0.06 (0.36)
To Test 6°	1.03 (0.42)	-0.01 (0.26)

To test whether the Anti-Test oriented arrows and the To Test oriented arrows differed from the Neutral Cue condition we computed their Δ d′. One-sample *t*-tests revealed that only the Anti-Test arrow orientations lead to significantly greater sensitivity compared to the Neutral Cue condition [Plus orientations: *t*_(14)_ = 3.5, *p* = 0.002, Cohen’s *d* = 0.89; Minus orientations: *t*_(14)_ = 0.4, *p* = 0.35; **Figure 5C**]. Bayesian one-sample *t*-tests found the Anti-Test orientations Bayes factor to be 7.31 (error % = 1.27e-5), indicating a moderate evidence that the sensitivity in these orientations differ from the Neutral Cue condition. The To-Test orientations Bayes Factor is 0.17 (error % = 2.52e-4), indicating a moderate evidence that the sensitivity in these orientations do not differ from the Neutral Cue condition.

The decision criterion in all four arrow orientation configurations was also calculated (see **Table 1** for criteria Means and SDs) and one-sample *t*-tests demonstrated that these did not defer from zero [Anti-Test 12°: *t*_(14)_ = 0.47, *p* = 0.64; Anti-Test 6°: *t*_(14)_ = -0.75, *p* = 0.46; To Test 6°: *t*_(14)_ = 0.42, *p* = 0.68; To Test 12°: *t*_(14)_ = -0.16, *p* = 0.88]. Hence, it appears that subjects‘ judgments were not simply biased toward the CW or the CCW direction under any configuration. To see if the orientation of the arrow affected not only the sensitivity, but also the criterion of the subjects, a Friedman test comparing the decision criteria in the four arrow orientations was conducted. No significant difference was found [χ^2^_(3,_ *_N_*_=__15)_ = 0.68, *p* = 0.88] indicating that the orientation effect observed was probably due to a change in sensitivity without any change in criterion. These results were validates by a Bayes Factor repeated measures ANOVA revealing that the null model was preferred to the model containing the Cue main effect by a Bayes Factor of 0.22 (error % = 0.58). The data provide moderate evidence for the hypothesis that the criterion was the same across all cue orientations. Bayesian one-sample *t*-tests moderately supported the hypothesis that the criterion in all four cue orientations did not defer from zero, providing evidence to a lack of bias toward CW or CCW responses in all orientations (To Test 12°: Bayes factor = 0.27, error% = 2.84e-5; To Test 6°: Bayes factor = 0.31, error% = 3.12e-5; Anti-Test 6°: Bayes Factor = 0.33, error% = 3.41e-5; Anti-Test 12°: Bayes Factor = 0.29, error% = 2.99e-5).

#### Neutral Cue and No Cue Conditions

Could the above results be due to the mere presentation of an interfering stimulus between Sample and Test, which modulated the sensitivity of the motion signal in WM? We argue that this is unlikely: if the arrow was simply disrupting the fidelity of the memory trace, it should have affected performance similarly in all arrow conditions, *independent of the arrow’s specific orientation*. This was clearly not the case. Furthermore, no significant difference in performance was found in a direct comparison between the Neutral Cue and No Cue conditions (Neutral Cue *Mean* = 1.18, *SD* = 0.45; No Cue *Mean* = 1.22, *SD* = 0.38; Wilcoxon Signed-ranks test *Z* = 0.28, *p* = 0.78), indicating that a non-oriented stimulus was not significantly disruptive in our testing conditions (**Figure 5B**). Furthermore, a Bayesian paired sample *t*-tests testing d′ in the Neutral Cue and No Cue conditions found a Bayesian Factor of 0.21 (error% = 2.528e-4), indicating the data supports moderately the null hypothesis over H1, i.e., that there is no general interference effect on delayed direction discrimination threshold.

We conclude that presentation of an oriented arrow in the delay period had biased the direction of the remembered motion. We suggest that this is because the memory trace of the motion stimulus is probably transformed into a more abstract form during the delay period, and is thus maintained in memory for future comparison.

## Discussion

The aim of this study was to gain better understanding of the nature of the representation of motion direction in WM. To that end, we conducted three experiments, testing different aspects of the mnemonic representation of motion.

### Experiment 1

First, we manipulated the Sample and Test stimuli brightness polarity, such that in some cases the SP was maintained while in others it was switched. We found no effect of contrast-polarity on performance threshold or slope, suggesting that the encoding and comparison of motion direction is done at a stage in which information from the separate On and Off pathways had been combined to yield a representation that is independent of stimulus polarity. These conclusions are based on not rejecting the null hypothesis and were confirmed by Bayesian analysis. Human psychophysical studies indicate that motion-processing mechanisms are sensitive to the polarity of motion signals. (Shechter and Hochstein, 1990; Wehrhahn and Rapf, 1992; Edwards and Badcock, 1994; Van Der Smagt and Van De Grind, 1999; Westheimer, 2007; Wuerger et al., 2011). For example, using random-dot kinematograms, Edwards and Badcock (1994) have shown that no motion is seen when a dot changes its polarity in a stroboscopic displacement experiment. This indicates that On and Off signals are analyzed separately during *local* motion processing. However, when extraction of the *global-motion* signal was required, the results indicated a sub-threshold *summation* of white and black dots, implying that at that stage, integration of information from the two streams had taken place (Edwards and Badcock, 1994).

Contrast-polarity sensitivity along the visual processing hierarchy was explored in several monkey studies, using an On-channel blocking agent (APB) that showed that the On and Off channels remain fully independent until the lateral geniculate nucleus (LGN) (Schiller, 1984). Studies conducted on primates and cats have shown that primary visual cortex input from each stream reaches spatially adjoining zones to generate a receptive field profile containing both On and Off subregions (Martinez et al., 2005; Mata and Ringach, 2005; Priebe and Ferster, 2005). At later cortical processing stages, (i.e., the MT and the MST) neurons respond to both types of stimuli with equal effectiveness (See Zeki, 1974; Tanaka and Saito, 1989; Clifford and Ibbotson, 2002 for review).

In our case, performance was unhampered by a change in dot contrast-polarity, confirming that it is based on a representation in which the On and Off pathways have been fully integrated.

### Experiment 2

In this experiment, we tested whether performance is affected by a shift in the spatial location of the two stimuli to be compared, in various coordinate frames. Specifically, we assessed performance level when the Sample and Test shared the same retinotopic or spatiotopic locations, or when they were located in different positions (either in the same hemifield or in opposite hemifields). We found no significant differences in threshold across all four different conditions that together with a Bayesian analysis, providing more evidence for this hypothesis, suggest that the comparison between motion signals is generalized across different spatial locations. The tolerance to changes in spatial location, in either coordinate frame, suggests that comparison is achieved by neuronal populations that receive motion information from wide parts of the visual field (more than 3.5° at 5° eccentricity, which is the separation between adjacent apertures in our experimental display). One possibility is that performance is based on higher-level cortical region, in which the receptive fields are large enough to cover the central parts of the ipsilateral visual field. Another, arguably more likely possibility, is that in humans, motion direction can be encoded and compared in a new format altogether (e.g., abstract representation) which is spatially invariant.

Non-retinotopic mnemonic representation was previously found using biological movement pattern stimuli (Wood, 2010). Subjects viewed first short video clips of people performing different movement patterns from different viewpoints and then viewed a second series of clips. They had to report which of the movements and/or viewpoints in the second series was presented before. The subjects’ memory capacity was independent of the viewpoint from which the movements were presented, and the information about the viewpoint from which each movement was observed was not readily accessible in memory. Thus, movement representations contain little to no view-dependent information, which suggests that VWM uses a non-retinotopic reference frame to retain movement information (Wood, 2010), in line with our own findings on non-biological motion.

It was also found that visual WM for sequentially presented patterns was insensitive to spatial displacements (when ISI was longer than 100 ms) during fixation (Phillips, 1974) or with an intervening saccade (Irwin, 1991). Moreover, Brockmole and Wang (2003) and Brockmole and Irwin (2005) have shown that the representation in visual WM is insensitive to other spatial properties, i.e., orientation and scale, and therefore may be abstract in nature. In their study, subjects viewed two sequentially presented 4 × 4 grids with dots occupying the grid-cells. The two grids differed in their orientation or in their scale, creating spatial mismatch between them. Subjects had to integrate the information from the two grids in order to report which grid-cell was empty across the two stimuli (Brockmole and Wang, 2003). Performance did not decrease due to the change in grid-orientation or scale, showing that the memory trace of the first stimulus was not spatiotopically or retinotopically specific; rather, subjects kept an abstract mnemonic trace of the grid. The abstract representation enabled it to be transformed to accommodate spatial changes, thereby providing spatial flexibility in the integration process of the two grid stimuli (Brockmole and Wang, 2003; Brockmole and Irwin, 2005).

Surprisingly, recent data showed that information about a remembered stimulus was kept in early visual cortex in a spatially generalized manner (both in the ipsilateral and contralateral cortices). Ester et al. (2009), asked subjects to remember the orientation of a grating presented in the left or right visual field. The specific fMRI activation patterns in V1 during *the delay period* (i.e., memory maintenance) allowed classification of the stimulus to be remembered with reasonable accuracy (e.g., well above chance level). Crucially, this information was available not only in voxel populations from the contralateral hemisphere, whose receptive fields match the position of the visual stimulus, but also when information was based solely on the activation pattern in the *ipsilatera*l hemisphere (Ester et al., 2009). A recent study by Pratte and Tong (2014) used a similar approach and showed that when the task required the binding of feature information to a specific location, areas V1 and V2 of the contralateral hemisphere carried more information regarding the remembered grating identity than their ipsilateral counterparts. In contrast, higher extrastriate areas exhibited similar levels of performance across the two hemispheres (Pratte and Tong, 2014). These results suggest that the spatial specificity of cortical mnemonic representations can be modulated by task demands.

Still, WM encoding in early visual cortex is highly contentious. Contrary to the findings described above and in other fMRI studies (Ester et al., 2009; Riggall and Postle, 2012, 2013; Emrich et al., 2013; Pratte and Tong, 2014), WM related activity in early visual cortex was recently called into question in a study by Mendoza-Halliday et al. (2014), at least for motion stimuli. Spiking activity recorded in the macaque MT region did not show any WM sustained activity related to the memorized motion direction, whereas the activity in higher visual areas, MST and LPFC, did show robust spiking activity related to WM. Reliably decoding the memorized direction of motion from the population spiking activity was possible only in MST and LPFC regions but not in MT area. The sustained activity sharply emerged as a *de novo* property of MST neurons, suggesting that along the dorsal visual pathway, the transformation of sensory representations of motion direction into mnemonic representations occurs in the MST circuitry. The contradiction between the fMRI literature and this study may be reconciled by the finding that information about the memorized direction of motion was present in local field potential (LFP) activity, recorded during the delay period in macaque MT region (Mendoza-Halliday et al., 2014). These oscillations in MT were phase-coherent with LPFC spikes, suggesting that the encoded memorized direction emerged from a top–down activation from the LPFC area (Mendoza-Halliday et al., 2014). These oscillatory signals were possibly the basis for the information decoded from the fMRI signals in early visual cortex regions (Logothetis et al., 2001; Magri et al., 2012). Our results are in line with these findings as we did not find evidence for sensory coding of motion which is believed to be represented in low level visual cortex (e.g., MT).

Our results are at odds with several studies reporting that performance of delayed motion discrimination tasks is superior when the stimulus is maintained in the same position: retinotopic or even spatiotopic location (Zaksas et al., 2001; Ong et al., 2009; Ong and Bisley, 2011). For example, a study using a delayed motion-direction discrimination task in monkeys, found that sensitivity dropped as the spatial separation between the two comparison stimuli increased, in a manner which closely matched the dependence of receptive field size on eccentricity in area MT (Zaksas et al., 2001; However see Felleman and Kaas, 1984, for larger receptive field size in area MT). These results suggested that task performance is determined by the activity of neuronal populations within MT. In another human study, using a delayed direction discrimination task (with an intervening eye movement as in our case), Ong et al. (2009) found that performance was better when the Sample and Test stimuli appeared in the same location on the screen, than when they appeared in the same retinal location. This incongruence with the results reported here might be due to several differences in the experimental design such as cue type, aperture shape or the specific motion directions used (main cardinal and oblique directions in Ong et al.’s (2009) design and non-principle directions here). However, the cause may lie in one important difference between all these studies and the one presented here: the nature of the particular discrimination subjects were performing. In the above mentioned studies, subjects were asked to determine whether the Sample and Test stimuli were the same or different (Zaksas et al., 2001; Pasternak and Zaksas, 2003; Ong et al., 2009), whereas in our study subjects had to compare the relative direction of motion (i.e., report whether the direction motion in the Test was CW or CCW relative to the motion in the Sample). We suggest that the usage of CW/CCW discrimination here required storage of the Sample stimulus for future comparison, and may have automatically prompted a more abstract mnemonic representation to encode the remembered motion direction (see Experiment 3 for further discussion).

### Experiment 3

In a third experiment, we followed the logic that the aspects of the remembered stimulus representation can be revealed, if task performance is affected by the presence of a task-irrelevant stimulus, shown during the memory delay period (Magnussen, 2000; Pasternak and Greenlee, 2005). It was previously shown that visual memory for simple features is vulnerable only to distractors containing conflicting information along the relevant stimulus dimension (Rademaker et al., 2015). For example, visual memory for spatial frequency was found to be immune to task-irrelevant interference by orientation (Magnussen et al., 1991; Lalonde and Chaudhuri, 2002; Nemes et al., 2011). Interference was explain in a model as a result of a lateral inhibition between specific higher-level modular memory stores in the visual cortex, which combine information from V1-type neural representations (Magnussen, 2000). According to this model, each dimension-dedicated module is organized as an array of memory stores that are linked in a lateral inhibitory network, and that each store codes a restricted range of values along that specific dimension (Magnussen, 2000). Based on these findings concerning interference specificity, if a mnemonic trace is vulnerable to interference by a distractor, then the trace and the distractor probably share some common features.

We found that the sensitivity to motion direction in a delayed memory task was predictably modulated by an arrow-shaped cue varying in its orientation. Task performance was better when the arrow was oriented away from the Test stimulus (thus increasing the perceived Sample-Test direction difference), than when it was oriented toward the Test’s direction (thus decreasing that difference). These results demonstrate that the mnemonic representation of motion is prone to interference by a pictorial task-irrelevant stimulus, and lead to the hypothesis that the memory trace of the Sample’s motion direction is represented not only sensorially, but also in an abstract form. We suggest that the Sample motion direction is transformed in memory into a pictorial (e.g., a line whose orientation is parallel to the motion direction) or even a more abstract mnemonic representation (but may still be represented in parallel by other more analog representations). This memory trace is biased by the arrow present in the delay period, such that it is attracted toward the arrow’s orientation. Hence, depending on the orientation of the arrow with respect to the Test stimulus, this arrow-based bias increased or decreased the difference between the Sample trace and the upcoming Test stimulus direction, and consequently amplified or reduced sensitivity, respectively.

Using an abstract mnemonic representation for motion direction seems logical when considering the specific nature of the discrimination task used in this study, namely CW versus CCW discrimination. We learn to associate direction with the position of the clock-hands. For example, the clock position can be used as an analogy to describe the relative position or direction of objects (Merriam-Webster.com, http://www.merriam-webster.com/dictionary/o’clock). The effect of learning to associate symbolic shape to a specific motion direction was studied by Schlack and Albright (2007) using electrophysiological recordings in monkeys. The authors trained monkeys to associate upward and downward pointing static arrow shapes with upward and downward moving dot arrays (Schlack and Albright, 2007). Prior to the associative training, neurons in MT did not respond selectively to the arrow shapes. Following training, a population of MT neurons showed an unprecedented selectivity for arrow direction corresponding to that predicted from the associated motion stimuli (Schlack and Albright, 2007). According to the authors, this activation is the neuronal embodiment of pictorially recalled motion (Schlack and Albright, 2007). In an analogy, when a CW/CCW discrimination is required (as in our case), the clock-hand representation might be automatically activated to encode the remembered motion direction (without being explicitly called for), thereby making it amenable to the oriented arrow-cue interference.

#### Ruling Out Alternative Accounts

In the next section, several alternative explanations at different stages of the performance of the task are discussed.

##### Repulsion effect between the Test and Sample stimuli

The perception of two motion directions can be biased in opposite directions. This phenomenon was demonstrated by Marshak and Sekuler (1979) who showed that when two random dot arrays are simultaneously presented, their directions of motion mutually repulse each other. Recently, Kang et al. (2011) have shown that this repulsion occurs even between a remembered and perceived directions of motion. Similar repulsion was also reported between the orientations of remembered and perceived Gabors (Scocchia et al., 2013). Such repulsion might have also occurred between our Sample and Test stimuli, exaggerating the difference between the Sample mnemonic trace and the perceived Test stimulus. However, in our case this repulsion was identical across all experimental conditions, as the direction shift between Sample and Test was of a constant 9° and therefore could not account for the variability in the pattern of our results.

##### Effects of the cue on the perception of the Test stimulus

A potential explanation could be that apart from affecting the memory trace of the Sample, the cue could also affect the perception of the upcoming Test. Like in the case of the repulsion between two motion stimuli, the arrow-shaped cue could repulse the Test, making its motion seem directed farther away from the orientation of the arrow. Assuming that the repulsion resembles what is known about motion stimuli (Rauber and Treue, 1999), this would predict better performance in the Anti-test cues (where the orientation of the cue and the Test direction of motion are 21° and 15° apart), and to a lesser extent in the To Test 6° cue (3° between cue orientation and Test direction). In this case, the repulsion would cause the Test direction to be perceived as farther away from the motion direction of the Sample. Conversely, we would expect a small detrimental effect in the To Test 12° cue (3° between cue orientation and Test direction, but in the opposite direction to the Sample), causing the Test to be perceived as closer to the direction of the Sample’s motion. However, the observed pattern of results is not consistent with this prediction (See **Figure 5**); suggesting that repulsion of the Test stimulus by the cue alone cannot explain the results.

Yet, it is possible that the repulsion of the Test by the Cue occurs on top of the effect of the Cue on the mnemonic trace of the Sample, adding up both effects. In this case, the linear sum of the two effects would yield the following pattern of results: a decline in performance in the To Test 12°, a smaller decline or no effect in the To Test 6°, an increase in performance in the Anti-Test 6° and a larger increase in the Anti-Test 12°. Based on the data presented here, this prediction cannot be refuted easily, but still, some theoretical and empirical considerations render it less probable. First, a repulsion of the Test stimulus by the Cue implies an interaction between stimuli from different classes, an arrow (a form stimulus) and a random-dot array, which are presumably processed in the ventral and the dorsal streams, respectively (see for review Ungerleider and Pasternak, 2004). Is repulsion between such different stimuli possible? It is becoming clear, that the interaction between form and motion processing is more extensive than previously thought (see for review Mather et al., 2013). There is evidence for interaction between orientation signals and motion in early visual cortex (V1 and V2) and even as high as areas MT, MST and STS (Mather et al., 2012, 2013; Pavan et al., 2013). However, there are several reasons that such direct perceptual interaction could not explain the effect described here. First, as the cue and Test stimuli are separated by 1200 ms, visually evoked motion and orientation signals must be preserved within a very long time-window for a direct perceptual interaction to happen. To the best of our knowledge, this has not been shown yet. Furthermore, empirical evidence suggests that there is no repulsion between an oriented line and a motion stimulus, presented sequentially, as are the cue and Test stimuli in our experiment. Kang et al. (2011, Experiment 5), using experimental configuration resembling much the setting used here, showed that a clock-hand in itself had no effect on the perception of a subsequent moving dot array’s direction. In their study, participants performed a dual task: they were presented with a clock-hand (an oriented line) whose orientation had to be memorized for a later recall, and then saw a moving dot array. They had to judge whether the dot array was moving CW or CCW relative to a reference bar. The perception of the dots’ motion direction was *not* biased by the preceding clock-hand, thus an oriented line does not affect the perception of a subsequent motion stimulus (Kang et al., 2011, Experiment 5). It seems likely therefore, that in our study, the perceived direction of the Test stimulus was not biased directly by the arrow-cue preceding it. Rather, the cue affected solely the memory trace of the Sample stimulus.

##### Attentional effects

Our results could be explained by an attention effect. A simple interference in attention could not explain the exact nature of biases discussed here, as also demonstrated by the Neutral Cue control condition, suggesting the involvement of a more specific process. Alternatively, the cue could have served as an attractor of feature/object-based attention. As such, the cue would facilitate the perception of congruent Test stimuli, i.e., stimuli with motion directions matching the arrow’s orientation, and hamper the processing of incongruent stimuli, i.e., stimuli with motion direction far from the arrow’s orientation. Here, the congruent cues are the To Test ones, in which the arrow is closer to the Test’s direction of motion, and the incongruent cues are the Anti-Test ones, in which the Test direction is farther away from the arrow’s orientation. Thus, a feature/object-based attention effect would predict better performance in the To Test cues than in the Anti-Test cues. However, our results show an opposite pattern: Anti-Test cues lead to larger d’ than To Test cues. Therefore, this is probably not such attention effect.

##### Use of strategies and decision stage effects

One may posit that the participants ignored completely the Sample stimulus and judged the direction of the Test relative to the *arrow-cue*. However, since in half the trials the arrow was pointing 12° away from the sample stimulus, *beyond* the Test motion direction, performance should have been around chance level, had participants adopted such a strategy.

Subjects could have also possibly based their answer on a direct comparison between the Sample and the arrow, ignoring the Test completely. Since the probability of the To-Test arrow orientations was 66.6% (see methods Oriented Cue Condition section), such a strategy would have led to higher performance on these arrow conditions than in the Anti-Test orientations. However, performance was higher in the Anti-Test than in the To-Test orientations (78 and 73% correct respectively). We conclude that the use of this alternative strategy is highly unlikely.

Potentially, subjects could also have adopted a two-step strategy, using the relative positions of the Sample, cue and Test stimuli to yield the correct response on all four cue orientations, without directly comparing the Sample and Test motion directions. According to such a scheme, at the first step subjects decided whether the cue orientation was CW or CCW with respect to the Sample’s direction of motion. On the second step, they judged if the cue’s orientation was close or far away from the Test’s motion direction. A lookup table giving the correct CW/CCW response for all four orientations can be formulated using the conjuncture of these two judgments (e.g., CW and far = CCW response; CW and near = CW response). However, this strategy provides equal information on the correct response in all four arrow orientations and therefore would predict a high and uniform performance across all orientations. This clearly could not explain the difference between the conditions that is the focus of this study. Moreover, this strategy is quite complex. Nine of the 15 subjects participated also in Experiment 1 and were therefore experienced in directly comparing the Sample and Test motion directions without any cue being present, so it is less likely that they had changed the way they performed the already learned task, switching to a more complicated strategy. Interestingly, the participants subjectively reported being able to compare the Sample and Test while ignoring the cue, and erroneously reported that the motion direction shift between the Sample and Test stimuli was varying, when veridically it was always constant. Taken together, this alternative explanation of strategy use is likely to be ruled out.

Finally, it is possible that the cue affected the decision stage and not the memory representation of the Sample motion. However, the decision criterion did not significantly differ across cue orientations and was centered near zero. These results are further supported by Bayesian analyses, suggesting there was no cue related change in the inclination to give a CW or CCW response.

#### The Attraction Effect

The symbolic cue shifted the sample memory trace toward the oriented cue. A similar attraction effect was described between the memory trace of a single randomly orientated grating and an interfering to-be-ignored distractor grating (Rademaker et al., 2015). A target oriented grating was first shown, followed by a 3 s delay during which a second, task-irrelevant grating, was presented. Subjects were instructed to memorize the first grating and adjust a probe grating to its remembered orientation and ignore the second one. Rademaker et al. (2015) found that the remembered orientation was biased toward the interfering grating by ∼3° and that larger differences between the memorized and distractor orientations led to noisier memory traces. Additionally, memory attraction was reduced when the interfering information was made task relevant, and memory biases were completely abolished when subjects did not consciously perceive the distractor when it was presented using a dichoptic mirror (Rademaker et al., 2015). The authors concluded that memory for orientation is prone to attraction bias that operates probably at the later stages of visual processing, and use a dynamic neural network model approach to tentatively explain these biases. According to this approach, the combination of delay-period dynamics (such as interactions between memory items presented at different times, different spatial locations, and between stored information and perceptual input) with consequences from newly arriving sensory input may add a subtle peak of activation in a neural model’s memory layer. If a small peak of distractor-centered excitation merges with the maintained representation one would expect a memory shift toward the distractor, as well as an increase in variance (Rademaker et al., 2015). The resemblance of this attraction bias between two oriented gratings to the attraction between motion and the arrow-shaped cue that we report here suggests that the orientation aspect of the arrow-shaped cue might be a relevant feature, contributing to our interference-induced bias.

Another example of an attraction effect was reported in the spatial frequency domain, between the remembered trace of a Gabor stimulus and a task-irrelevant non-target Gabor accompanying it (Huang and Sekuler, 2010; Dubé et al., 2014). In Huang and Sekuler’s (2010) study, participants sequentially saw 2 Gabor stimuli differing in their spatial frequency. A cue was presented either before or after their presentation, indicating which of the two Gabors’ spatial frequency should be reported later (by adjusting a probe Gabor). When the cue was shown *after* the Gabors‘ presentation, such that encoding was performed under uncertainty regarding task relevance, the reported spatial frequency was biased toward the non-target Gabor’s frequency (Huang and Sekuler, 2010). However, when uncertainty was reduced (by indicating which of the 2 Gabors was task-relevant before their presentation) this attraction almost disappeared (Huang and Sekuler, 2010). The authors suggest that the attraction is due to perceptual averaging: subjects stored a weighted average of the relevant and irrelevant memory items which subsequently influenced recall of the relevant item (Dubé et al., 2014). This is thought to be a neural process happening under conditions of uncertainty about the contents of visual short term memory, in which spatiotemporal activation patterns evoked by the presentation of multiple stimuli within the receptive fields of different neurons, approximate an average of the neural responses evoked by the stimuli individually (Huang and Sekuler, 2010; Dubé et al., 2014). There are a few differences between the results of these studies and our findings. First, Huang and Sekuler (2010) and Dubé et al. (2014) reported attraction biases mainly under uncertainty regarding task relevance, yet we found an attraction effect between the cue and Sample even when subjects knew in advance which of the two stimuli was relevant to the task. Additionally, the neural mechanism suggested to perform the perceptual averaging involves activation within the receptive fields of neurons by multiple stimuli *of the same kind* (i.e., Gabors). As discussed above (see Repulsion Effect Between the Test and Sample Stimuli), in our case, the attraction is between stimuli from different classes, an arrow (a form stimulus) and a random-dot array, which were shown to be processed in the ventral and the dorsal streams (Ungerleider and Pasternak, 2004). As argued before for repulsion, direct interaction between such different stimuli, which is required for perceptual averaging, is not likely to explain the attraction effect described here. Both the long time interval between the presentation of the stimuli and empirical evidence showing no direct perceptual interaction between sequentially presented orientation and motion stimuli (Kang et al., 2011, Experiment 5), argue against such explanation. Thus, we think that neuronal averaging at the perceptual level is unlikely to be the basis of the attraction shown here.

Whatever the mechanism underlying the attraction effect mediated by the abstract cue, it is possible that it *interacts* in a non-linear way with the repulsion between the Test and Sample motion stimuli, and might possibly explain the asymmetrical effect in respect with the No Cue control. Specifically, the finding that only the sensitivity in the Anti-Test cue orientations differed significantly from the Neutral Cue condition, could potentially result from a supra-linear summation of the two effects when they are yielding a similar bias in perception (increase of the direction difference between the Sample and the Test); and/or a sub-linear effect when repulsion and attraction operate in opposite directions. This point remains to be discovered in future research.

Were the repulsion and attraction operating on a single mnemonic trace? It has been proposed that multiple representations are stored in WM (Postle et al., 2005; Brady et al., 2011; Dubé et al., 2014). Thus, we suggest that there might be at least two parallel mnemonic traces during the delayed direction discrimination task: a low-level sensory trace representing motion in an analog form and subject to mutual repulsion, and a higher-level abstract representation susceptible to attraction. What is the neural basis that supports these representations, and their precise nature is yet to be seen.

Another key point that merits additional examination concerns the exact nature of the representation of motion in WM: Is the suggested memory representation of the motion direction elicited by the CW/CCW discrimination task a pictorial symbolic representation (i.e., an oriented “clock-hand-like” line), or does it include in addition a verbal component? Could a verbal cue bias the delayed discrimination as did the arrow? In this context it is important to note that we do not refer here to an explicit verbal encoding of the motion stimuli during perception. The two most prominent verbal encoding strategies: using the hours of an analog clock and naming the cardinal directions, were made improbable in our set-up of Experiment 3 because the direction differences used were much smaller than the hours in an analog clock (8° difference between the different Sample directions and 9° difference between Sample and Test stimuli) and excluded the cardinal directions and main diagonals. Thus, if a verbal component participates in the mnemonic representation of motion in this task it is probably activated implicitly after the perception stage.

To summarize, we present here psychophysical evidence that the representation of motion direction in WM is insensitive to dots’ brightness polarity and is spatially invariant. This mnemonic trace is predictably biased toward the direction of an arrow shaped cue suggesting that an abstract motion vector-like trace, encoding the direction of motion (maybe somewhat analogous to an arrow), is maintained in WM. It is plausible that such an abstract mnemonic trace might be activated alongside with a more basic, analog representation of the stimulus. We speculate that the specificity of this abstract representation to an arrow-shaped symbol may stem from the long learned association of motion direction and clock hand pictorial symbols.

## Author Contributions

TSM designed the experiments, programmed and ran them, analyzed the data, and wrote the paper. YP programmed one of the experiments and ran it, and wrote the paper. EZ designed the experiments, analyzed the data, and wrote the paper.

## Conflict of Interest Statement

The authors declare that the research was conducted in the absence of any commercial or financial relationships that could be construed as a potential conflict of interest. The reviewer BP and handling Editor declared their shared affiliation, and the handling Editor states that the process nevertheless met the standards of a fair and objective review.
